# Identification of a novel Leucine-rich repeat protein and candidate PP1 regulatory subunit expressed in developing spermatids

**DOI:** 10.1186/1471-2121-9-9

**Published:** 2008-01-31

**Authors:** Rong Wang, Ann O Sperry

**Affiliations:** 1 Department of Anatomy and Cell Biology, Brody School of Medicine at East Carolina University, Greenville, NC 27834 USA

## Abstract

**Background:**

Spermatogenesis is comprised of a series of highly regulated developmental changes that transform the precursor germ cell into a highly specialized spermatozoon. The last phase of spermatogenesis, termed spermiogenesis, involves dramatic morphological change including formation of the acrosome, elongation and condensation of the nucleus, formation of the flagella, and disposal of unnecessary cytoplasm. A prominent cytoskeletal component of the developing spermatid is the manchette, a unique microtubular structure that surrounds the nucleus of the developing spermatid and is thought to assist in both the reshaping of the nucleus and redistribution of spermatid cytoplasm. Although the molecular motor KIFC1 has been shown to associate with the manchette, its precise role in function of the manchette and the identity of its testis specific protein partners are unknown. The purpose of this study was to identify proteins in the testis that interact with KIFC1 using a yeast 2 hybrid screen of a testis cDNA library.

**Results:**

Thirty percent of the interacting clones identified in our screen contain an identical cDNA encoding a 40 kD protein. This interacting protein has 4 leucine-rich repeats in its amino terminal half and is expressed primarily in the testis; therefore we have named this protein testis leucine-rich repeat protein or TLRR. TLRR was also found to associate tightly with the KIFC1 targeting domain using affinity chromatography. In addition to the leucine-rich repeats, TLRR contains a consensus-binding site for protein phosphatase-1 (PP1). Immunocytochemistry using a TLRR specific antibody demonstrates that this protein is found near the manchette of developing spermatids.

**Conclusion:**

We have identified a previously uncharacterized leucine-rich repeat protein that is expressed abundantly in the testis and associates with the manchette of developing spermatids, possibly through its interaction with the KIFC1 molecular motor. TLRR is homologous to a class of regulatory subunits for PP1, a central phosphatase in the reversible phosphorylation of proteins that is key to modulation of many intracellular processes. TLRR may serve to target this important signaling molecule near the nucleus of developing spermatids in order to control the cellular rearrangements of spermiogenesis.

## Background

Spermatogenesis consists of three phases: mitotic division of spermatogonia, meiotic division of spermatocytes and cellular transformation of haploid gametes during spermiogenesis. The final phase requires quite striking cellular reorganization to produce functional sperm including biogenesis of spermatid specific organelles and structures such as the acrosome and microtubule manchette, removal of excess cytoplasm, and streamlining of the spermatid nucleus into its final shape [1]. These unique forms of intracellular motility are expected to require specific adaptations of the spermatid cytoskeleton and associated molecular motor proteins [2]. We have identified a kinesin-related molecular motor, KIFC1, a C-terminal motor and member of the Kinesin-14 subfamily [3], associated with the spermatid nucleus during the morphological changes of spermiogenesis [4]. This molecular motor is first seen adjacent to the round spermatid nucleus in early spermatids of approximately step 1–2 and then migrates to the nuclear surface and the growing acrosome of step 7–8 spermatids [4]. In more elongate spermatids, KIFC1 locates near the spermatid manchette, a spermatid-specific microtubule structure thought to be important for spermatid nuclear shaping [5] and redistribution of cytoplasm (reviewed in [6]).

Previous work identified a 19 amino acid sequence in the tail domain of KIFC1 that is necessary and sufficient to target this motor to membranous structures in cultured cells [7]. Furthermore, we have shown recently that this domain assembles a complex containing the nucleoporin NUP62 in testis lysate [8]. The localization of this molecular motor near the nucleus of elongating spermatids and its association with proteins of the nuclear membrane makes KIFC1 an excellent candidate for participation in the unique transformation of the nucleus that occurs during spermiogenesis. We have utilized the targeting sequence of KIFC1 to identify interacting proteins in the testis that may be important for this process. We describe here the identification of a testis-specific leucine-rich repeat protein that contains a docking site for PP1 and is localized near the nucleus of developing spermatids.

## Results

### A leucine-rich repeat protein interacts with the KIFC1 targeting sequence

**Figure 1:**
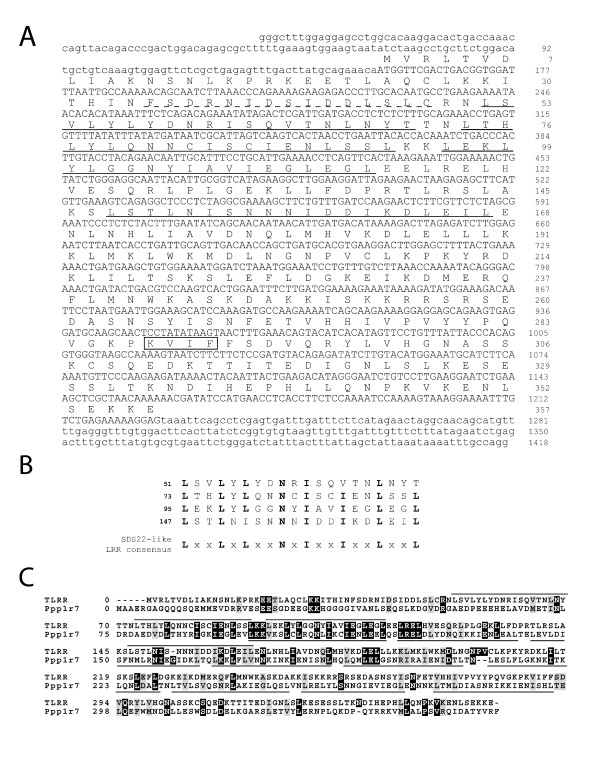
**TLRR is a leucine-rich repeat protein**. (A) The DNA sequence of the interacting clone (IC#2) is shown with the translated sequence above the nucleotide sequence. The leucine-rich repeats (LRRs) are indicated with solid underlining and the peptide used for preparation of antibody indicated with dashed underlining. The putative PP1 binding site is boxed. (B) The TLRR repeats are aligned with the LRR consensus sequence for the sds22 subfamily of LRR proteins. (C) An alignment between TLRR and Ppp1r7 is shown with the LRRs indicated with overlines (TLRR) and underlines (Ppp1r7). Multiple sequence alignment was done using ClustalW 1.82 and the results displayed using BOXSHADE 3.31. LRRs in TLRR and Ppp1r7 were identified using the Pfam protein domain database [35].

In an effort to identify proteins associated with the KIFC1 targeting domain, we used the 19 amino acid targeting sequence as bait to screen a mouse testis cDNA library using a yeast 2-hybrid approach [9]. Approximately 2 × 10^7 ^independent clones were screened for induction of transcription of three genes (HIS3, ADE2, and MEL1) by bait-prey interaction. Of the 30 positive clones identified, 10 were identical and contained an open reading frame for an uncharacterized protein previously identified in a mouse adult testis cDNA library (accession number AK076637). The approximately 40 kD predicted open reading frame contains 4 leucine rich repeats (LRRs) in the N-terminal half of the molecule (underlined in Figure 1A) and was therefore named testis leucine rich repeat, or TLRR. Three of the LRRs match the consensus repeat domain previously described for the sds22 subfamily of LRR proteins while the other is a slight variation (Figure 1B) [10,11]. The TLRR protein demonstrates 15% sequence identity and 27% homology to Ppp1r7 (Figure 1C), the mouse homolog of yeast SDS22p [12], a regulator of PP1; however, the homology is greatest in the regions containing the LLR domains. Sequence analysis revealed a variant of the consensus binding site for PP1, (R/K)x_1_(V/I)x_2_(F/W), in the carboxyl half of the polypeptide [13] (boxed sequence, Figure 1A).

### TLRR is expressed in the testis

**Figure 2:**
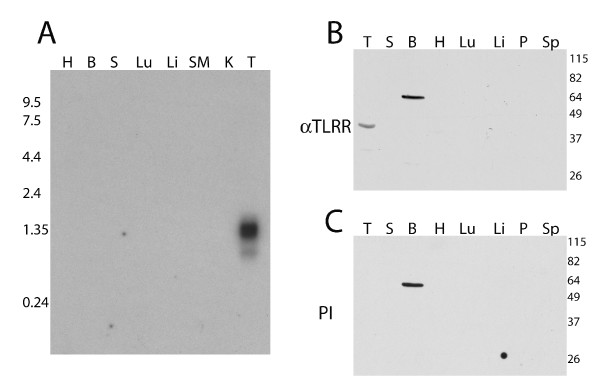
**TLRR is expressed primarily in testis**. (A) A multiple tissue northern blot was hybridized with radiolabeled probe specific for the TLRR mRNA as described in Methods. Each lane contains 2 μg mouse poly(A+) RNA separated on a denaturing formaldehyde 1.0% agarose gel and transferred to nylon. Lanes 1–8 contain: H, heart; B, whole brain; S, spleen; Lu, lung; Li, liver; SM, skeletal muscle; K, kidney; T, testis. The migration and size of RNA markers is shown on the left. (B and C) Total protein (75 μg) from each of the indicated tissues was separated by PAGE, transferred to membrane, and incubated with the affinity purified TLRR antibody (B), or preimmune serum (C). Lanes 1–8 in both B and C contain: T, testis; S, spleen; B, whole brain; H, heart; Lu, lung; Li, liver; P, pancreas; Sp, epidydimal sperm.

To determine whether TLRR is expressed in a tissue-specific manner, we tested for the presence of TLRR mRNA in various mouse tissues. TLRR is highly and, of the tissues we tested, exclusively expressed in the testis (Figure 2A), a pattern consistent with its assigned Unigene cluster at the NCBI, Mm.386795. Even upon longer exposure, no signal is detected in the other tissues examined. Two TLRR variants have been entered into available databases (accession numbers NM_027033.1 and NM_145692.1), perhaps explaining the diffuse nature of the band at around 1.7 kb. Interestingly, the shorter variant stops just after the last LRR and therefore does not contain the predicted PP1 binding site.

In order to detect the TLRR protein in tissue lysates and in sections, an anti-peptide polyclonal antibody was prepared specific for the TLRR protein (see Methods section). The TLRR specific antibody recognizes a 40 kD band only in testis lysate, a size that matches the predicted ORF of the interacting clone (Figure 2B). No protein of this size is detected with the TLRR antibody in the other tissues examined. A 64 kD band is evident in brain but is also recognized by preimmune serum (Figure 2C) and therefore likely represents a nonspecific epitope enriched in this tissue. No signal was visible in epididymal sperm lysate, even at longer exposures, suggesting that TLRR may be discarded during spermatogenesis.

### Interaction of TLRR with KIFC1 requires all LRRs

**Figure 3:**
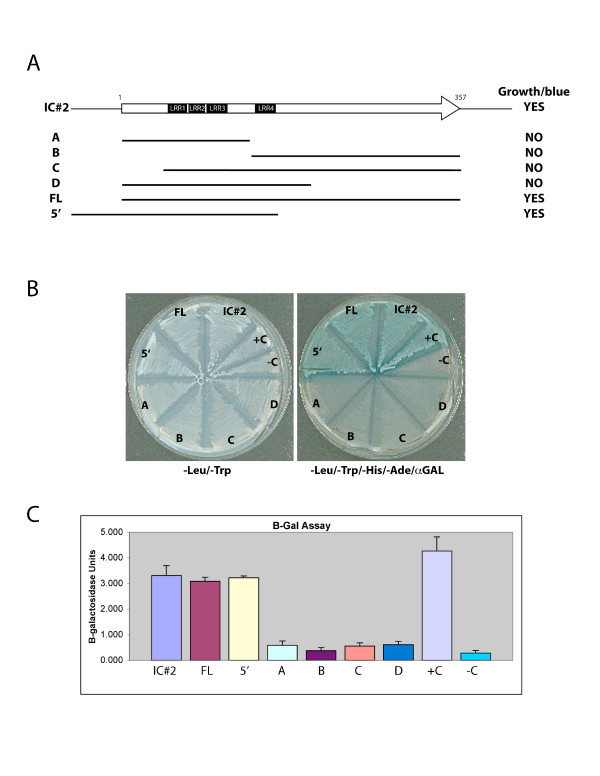
**LRR domains are not sufficient for interaction with KIFC1 tail sequence**. (A) A schematic is shown of the KIFC1 interacting clone with the 357 amino acid open reading frame indicated with an arrow. The LRR domains are indicated with black boxes and below are shown the regions tested for interaction with the KIFC1 bait plasmid and the results of interaction tests. (B) Plate assay of interaction of TLRR deletion constructs with KIFC1bait. The plate on the left selects only for yeast harboring both bait and prey plasmids; all cotransformants are able to grow. The plate on the right also lacks adenine and histidine and is therefore selective for transactivation of the reporter genes. Transactivation of the MEL1 reporter gene is detected by incorporation of X-α-gal into the media resulting in blue colonies. Positive control for protein interaction (+C) is yeast cotransformed with pGADT7-T and pGBKT7-53 while cells containing pGADT7 and pGBKT7-Lam (-C) is negative control. (C) Liquid assay of interaction of TLRR deletion constructs with KIFC1bait. The β-galactosidase activity of cultures of TLRR/KIFC1 bait cotransformants was determined as described in Methods. Each assay was repeated at least three times. Standard error of the mean for each transformant is indicated by bracketed lines in panel C.

In order to determine whether any or all of the LRRs in this protein contribute to it's binding to KIFC1, we constructed deletion mutants of TLLR followed by detection of protein interaction by yeast 2-hybrid. Fragments expressing portions of the TLRR 40 kD open reading frame were transferred from one of the original clones (IC#2) to pADT7 for expression in yeast (Figure 3A). Each construct was cotransformed with the KIFC1 bait plasmid into AH109 and protein interaction tested by growth on selective media containing X-α-Gal. Only the original clone (IC#2), the complete open reading frame (FL), and a construct containing sequences 5' to the putative ATG in addition to the LRRs, were able to support transcriptional activation in yeast (Figure 3B). Constructs containing less than the full complement of LRRs (constructs A and B) as well as those containing all four LRRs but truncated at either the amino (construct C) or carboxyl (construct D) where unable to transactivate. This suggests that all 4 LRR sequences are necessary but not sufficient for interaction with KIFC1. Surprisingly, the construct containing the 4 LRRs (lacking only the last leucine in LRR4) but containing coding sequence upstream of the putative ATG was able to activate transcription, perhaps indicating that the conformation of this construct (TLRR5') is more favorable for interaction than TLRRD. β-gal assays of liquid cultures of each yeast cotransformant are consistent with the plate assays (Figure 3C).

### TLRR is specifically associated with the KIFC1 targeting sequence

**Figure 4:**
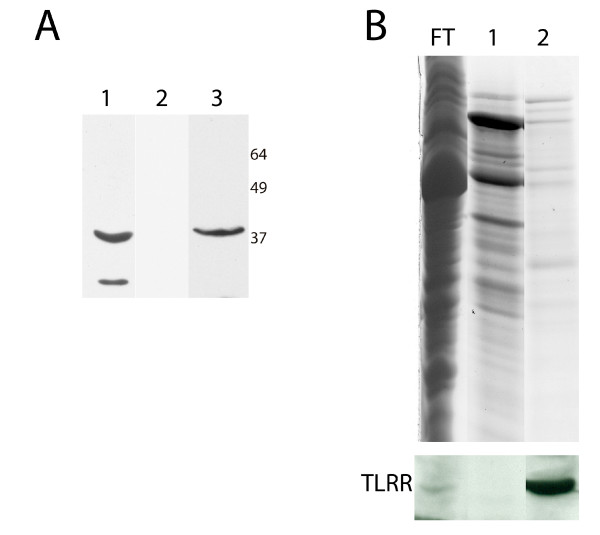
**TLRR associates tightly with the KIFC1 targeting sequence**. (A) 200 μg testis lysate (lane 1 and 2) or an aliquot of recombinant HIS6 tagged TLRR (lane 3) were resolved by SDS-PAGE and blotted with TLRR antibody (lane 1 and 3) or preimmune serum (lane 2). (B) Testis lysate was loaded onto a column consisting of KIFC1 targeting peptide-linked resin. Flow through (FT) was collected, the column washed and bound complexes eluted with glycine, pH 2.5 (lane 1) or glycine plus 0.5 M NaCl (lane 2). Coomassie stained gel is shown at top with TLRR western below.

We have previously used an affinity approach to purify testis proteins associated with the KIFC1 targeting domain and have shown that the rat TLRR protein is specifically bound to the KIFC1 affinity column [8]. We next wanted to determine whether mouse TLRR is specifically associated with the KIFC1 affinity column. In order to detect the TLRR protein in fractions bound to the affinity column we used our affinity purified TLRR antibody. This antibody reacts with a 40 kD band in testis lysate (Figure 4A lane 1) and with recombinant TLRR expressed in bacteria (Figure 4A lane 3). The TLRR specific antibody also recognizes a faster migrating band in testis lysate that could result from a shorter TLRR isoform, or could be due to proteolysis. No reactive band in testis was detected when the blot was probed with preimmune serum (Figure 4A lane 2). Testis lysate was incubated with the KIFC1 targeting peptide linked to resin and proteins present in the flow-through and eluted fractions were resolved by PAGE and visualized by staining and western blot with TLRR specific antibody (Figure 4B). A subset of lysate proteins was found to associate with the KIFC1 peptide and was eluted with low pH (Figure 4B, lane 1) or low pH plus NaCl (Figure 4B, lane 2). A band reactive to the TLRR antibody is detected in the low pH plus salt elution (Figure 4B, lane 2).

### TLRR is localized to germ cells in the testis

**Figure 5:**
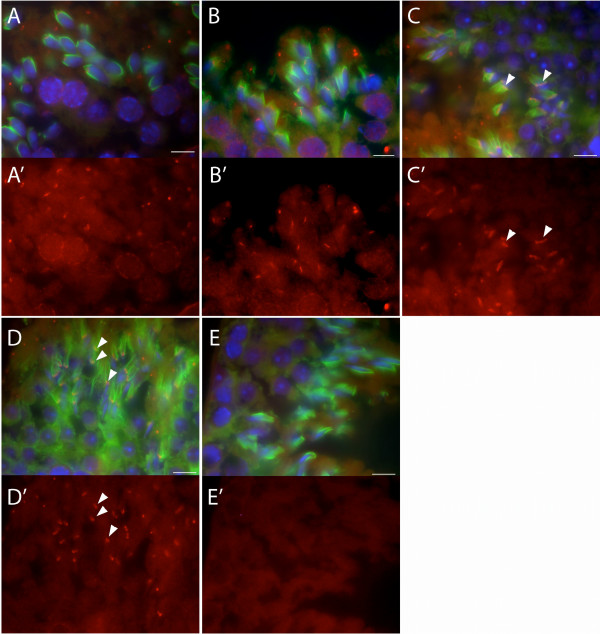
**TLRR localizes to the manchette of developing spermatids**. (A-D) Merged images of adult mouse testis sections triple stained with anti-TLRR (red), anti-α-tubulin (green, manchette), and DAPI (blue, nuclei) as described in the Methods section. (A'-D') TLRR signal corresponding to each of the merged images shown in A-D. Increasingly elongated spermatids are shown from panels A, A' through D, D'. TLRR is associated with the manchette in mid stage spermatids (arrowheads, panels C and C') and this staining narrows with the elongation of the manchette in approximately step 15 spermatids (arrowheads, panels D and D'). (E, E') Negative control for these experiments where TLRR specific primary antibody was omitted. Bar represents 10 μm.

The TLRR specific antibody was used to localize this protein in mouse testis sections. The TLRR antibody stains developing spermatids on or near the manchette (stained with anti-α-tubulin in Figure 5A–E) at different developmental stages. In early spermatids, when the manchette first appears, TLRR is localized adjacent to the spermatid nucleus (Figure 5A, 5A' and 5B, B'). In the more mature spermatids shown in Figure 5C and 5C' (approximately step 12), TLRR is found near the manchette (arrowheads, Figure 5C, C'). Just prior to dissolution of the manchette, in approximately step 15 spermatids, TLRR still resides near the manchette but has narrowed along with this structure (arrowheads, Figure 5D and 5D'). The TLRR antibody does not appear to stain the entire manchette but instead is restricted to a subdomain near the perinuclear ring. In addition to spermatid staining, we noted nuclear rim staining in pachytene spermatocytes (Figure 5A, A'). Only background signal was detectable in sections incubated without primary antibody (Figure 5E, E').

**Figure 6:**
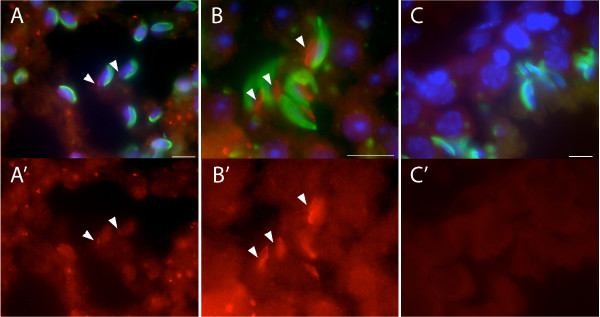
**TLRR localizes near the distal pole of the spermatid nucleus**. (A-B) Merged images of adult mouse testis sections triple stained with anti-TLRR (red), peanut agglutinin (green, acrosome) and DAPI (blue, nuclei). (A'-B') TLRR signal corresponding to each of the merged images shown in A-B. TLRR is found near the nuclear membrane at a site opposite the acrosome and is most prominent in later step spermatids (arrowheads, panels B and B'). (C, C') Control staining for this series where TLRR antibody was replaced with normal rabbit IgG. Bar represents 10 μm.

In order to determine the relative localization of TLRR on the spermatid nucleus with respect to the acrosome, testis sections were triple stained with TLRR antibody, DAPI and a FITC conjugated lectin, peanut agglutinin (Figure 6A–B). TLRR was first observed on the nuclear surface, opposite the acrosome in elongating spermatids of step 8–9 (arrowheads, Figure 6A, A'), followed by limited spreading over the distal portion of the nucleus at the position occupied by the manchette, in later spermatids of about step 12 (arrowheads, Figure 6B, B'). No signal was detected when the primary antibody was replaced with normal rabbit IgG (Figure 6C, C').

## Discussion

Testis leucine rich repeat, or TLRR, was identified as a protein interacting with the 19 amino acid targeting sequence of the KIFC1 molecular motor in a testis cDNA library. TLRR contains 4 leucine rich repeats (LRRs) in its amino terminus. LRRs are 20–29 residue sequence motifs that are predicted to form a horseshoe shaped structure with internal paired β-sheets and external helices. LRR containing proteins are proposed to function in many different biological processes by providing a versatile structural framework for protein-protein interactions (reviewed in [10]). The LRR sequences in TLRR most closely match those found in sds22, a regulatory subunit of PP1 in yeast [14]. Indeed, TLRR contains a consensus-binding site for PP1 in its carboxyl terminal domain (Figure 1A). PP1 is a key serine/threonine phosphatase that controls an extremely wide variety of biological processes. In contrast to mammalian kinases which are represented by many genes, the catalytic subunit of PP1 (PP1c) is encoded by just three genes in rodents: PP1α, PP1β/δ and PPγ [15]. The functional diversity of PP1 is achieved by interaction of the catalytic subunit with a variety of regulatory subunits that serve to modulate the activity of this enzyme and target it to the correct intracellular location and/or substrate (reviewed by [13,16,17]). TLRR is a good candidate for a testis-specific regulator of PP1 and may participate in regulation of the cellular transformations of spermiogenesis.

Most PP1 isoforms are expressed in a variety of tissues, except for PP1γ2, which is expressed primarily in testis [18]. PP1 isoforms are differentially expressed in male germ cells and mature spermatozoa [19] and one isoform, PP1γ2, is essential for male fertility [19,20]. The mammalian homolog of sds22 associates with PP1γ2 in bull spermatozoa where it negatively regulates the activity of this enzyme thereby modulating protein phosphorylation and sperm motility [21,22]. In contrast to sds22, we cannot detect TLRR in sperm lysate suggesting that TLRR does not play a role in regulating flagellar motility. Besides its presence in the flagella of mature sperm, PP1γ2 is present in differentiating germ cells including secondary spermatocytes, and round and elongating spermatids where it has been suggested to play a role in the morphogenesis of these cells [19]. TLRR is also expressed in these cell types, however, TLRR is localized near the spermatid nucleus and manchette (Figure 5 and 6), whereas PP1γ2 is expressed throughout the cytoplasm [19].

The manchette is a microtubule based structure that is unique to developing male germ cells. It forms around the nucleus of approximately step 8 spermatids and disappears by step 16. Although it has been studied for many years [23,24], its precise function and particularly the regulation of its motility along the nucleus are not completely understood. Disruption of the manchette by genetic or chemical means results in distortion of the underlying spermatid nucleus, suggestive of a role for the manchette in nuclear shaping [5,25]. The manchette has also been proposed to act as a track for the transport of cellular components from the apical to the distal end of the elongating spermatid and as a platform for the localization of signaling molecules [26,27]. The manchette is present at an optimal temporal and spatial position to provide a site for the assembly of signaling complexes necessary for regulation of the dramatic morphological transformation of the spermatid. Several potential regulatory proteins have been localized to the manchette including RanGTPase [28,29], a testis specific serine/threonine kinase [30], and NDP kinase [31]. We present evidence here that a candidate regulatory subunit of PP1 is also localized to this structure.

## Conclusion

The work presented here supports the idea that TLRR is a testis specific regulatory subunit of PP1 that might serve to target PP1 and perhaps other regulatory molecules to the nucleus of developing spermatids. TLRR is expressed primarily in the testis where it is localized near the manchette of developing spermatids, perhaps via its association with the microtubule motor KIFC1; both proteins are present at the same time and in a similar pattern on the spermatid nucleus [4]. TLRR is homologous to other PP1 regulatory subunits and contains a binding site for PP1. It will be important to determine whether TLRR physically associates with PP1, whether this association is isoform specific and whether TLRR is able to regulate the activity of this enzyme.

## Methods

### DNA manipulations and Yeast 2-hybrid screen

The KIFC1 bait plasmid was constructed by annealing two complementary oligonucleotides (KIFC1sense and KIFC1antisense) that encode the 19 amino acid targeting sequence followed by ligation into pGBKT7 at the EcoRI and BamHI sites. The sequence of KIFC1sense is GAATTCGGAAAGGCTGCTTCAGGAGCTTCAGGGAGAGCGGCTGCA ATTGCAGGAAGAGCGGACGGATCC and KIFC1antisense is GGATCCGTCCGCTCTT CCTGCAATTGCAGCCGCTCTCCCTGAAGCTCCTGAAGCAGCCTTTCCGAATTC. The sequence of the pKIFC1bait was confirmed by sequencing. The pKIFC1bait plasmid was transformed into yeast strain AH109 and mated to a pretransformed testis cDNA library (Clontech; Palo Alto, CA) in yeast strain Y187. Positively interacting clones were selected for growth on media lacking histidine, adenine, leucine, and tryptophan and containing X-α-Gal. Diploids were restreaked on selective media and the phenotype retested. The interacting prey plasmids were rescued from each clone according to the manufacture's instructions and used to transform competent cells (Invitrogen; Carlsbad, CA). Plasmid DNA was then isolated using the Wizard Plus SV miniprep kit (Promega; Madison, WI) sequenced by the DNA sequencing facility at ECU and the sequences compared against available databases. The TLRR deletion mutants were constructed by using appropriate primers to amplify the desired fragments by PCR before transfer into pADT7 using the restriction sites EcoRI and BamHI engineered into the PCR primers. TLRRFL (full-length) was amplified with GAATTCGTTCGACTGACGGTGGATTT AATTGCC as the 5' primer and GGATCCCTCCTTTTTCTCAGACAAATTTT CC as the 3' primer. The TLRRA fragment was amplified with the TLRRFL5' primer and GGAATCCCAGA AGCTTTTCGCCTAGAGGGAGCC as the 3' primer. TLRRB was amplified with the TLRRFL3' primer and GAATTCTTTGATCCAAGAACTCTTCGTTCTCTAGCG as the 5' primer. TLRRC was amplified with TLRRFL3' primer and GAATTCTGCAGAAACCTG AGTGTTTTATATTTA as the 5' primer. TLRRD was amplified with the TLRRFL5' primer and GGATCCAGGATTT CCATTTAGATCCATTTTCC as the 3' primer. The TLRR5' deletion mutant was constructed by partial deletion of the IC#2 interacting clone with BglII followed by religation and therefore contains sequences 5' to the ATG of the putative TLRR and extends through amino acid number 166 eliminating the last leucine of the fourth TLRR motif. For characterization of the TLRR antibody, the TLRR ORF was cloned into the bacterial expression vector QE31 in frame with the His6 tag.

### β-gal activity assay

β-Galactosidase activity was measured in liquid cultures of yeast using ONPG (O-nitrophenyl B-D-galactopyranoside) as substrate. Cultures of each yeast co-transformant were grown overnight at 30°C in 5 ml of SD selective medium (SD-LEU-TRP, Clontech; Palo Alto, CA), diluted with YPD medium (BD Bioscience; Bedford, MA) and growth continued at 30°C for 3–5 hours with shaking at 230 rpm until the cultures reached OD_600 _0.5–0.8. 1.5 ml of each culture was transferred to eppendorf tubes, centrifuged, and resuspended in approximately one fifth the original volume with Z buffer (0.06 M Na_2_HPO_4_, 0.04 M NaH_2_PO_4_, pH 7.0, 0.01 M KCl, 0.001 M MgSO_4_). Cells were broken by repeated freeze/thaw and the reaction started by addition of 0.7 ml Z buffer with 0.27% β-mercaptoethanol and 160 μl of ONPG (4 mg/ml O-nitrophenyl B-D-galactopyranoside, in Z buffer, Sigma; Atlanta, GA), followed by incubation at 30°C until reactions became yellow, and terminated with 0.4 ml of 1 M Na_2_CO_3_. The absorbance of each supernatant at OD_420 _was determined and the activity calculated according to the following equation: $\frac{1000\times OD420}{T\times V\times OD600}$, where T is the incubation time in minutes, V is 0.1 ml × concentration factor, and OD_600 _is the A_600 _of 1 ml of culture. The activity of each co-transfectant was assayed in triplicate. The yeast strain AH109 (Clontech; Palo Alto, CA) cotransformed with pGADT7-T and pGBKT7-53 were used as positive control for protein-protein interaction while cells containing pGADT7 and pGBKT7-Lam, expressing proteins that do not interact, were negative controls for these experiments.

### Northern Analysis

A normalized multiple tissue Northern blot (Clontech; Palo Alto, CA) was used to identify TLRR transcripts in different mouse tissues using the TLRRA fragment described above as probe (Figure 3A). Total RNA from various tissues was also isolated using the Trizol reagent (Invitrogen; Carlsbad, CA), separated, blotted to Nytran (Schleicher and Schull; Keene, NH), and probed with the TLRRA fragment giving identical results (data not shown). The DNA probe was labeled by mixed primer labeling using the High Prime kit from Roche Diagnostics (Indianapolis, IN) and [α^32^P]dATP. Blots were hybridized to probe in ExpressHyb solution (Clontech; Palo Alto, CA) or Church and Gilbert solution [32] overnight at 68°C. After hybridization, blots were washed at high stringency and exposed to X-ray film with a intensifying screens for 1 hour.

### Antibody Preparation and Western Blot

The TLRR sequence was scanned for antigenic peptides using the EMBOSS site [33] and a unique peptide, FSDRNIDSIDDLSLC, was chosen to immunize rabbits (Harlan Bioproducts for Science; Indianapolis, IN). The resultant sera were affinity purified against the corresponding peptide using the Sulfolink kit (Pierce; Rockford, IL) according to the manufacturer's instructions. Protein samples containing equal protein prepared from mouse tissue extracts, or affinity purification fractions, were separated by polyacrylamide gel electrophoresis (PAGE) through 10% acrylamide gels or precast 8–16% acrylamide gels (Invitrogen; Carlsbad, CA), equilibrated in and electrophoretically transferred from the gel matrix to PVDF membrane (BioRad Laboratories; Hercules CA) in Towbin transfer buffer. Proteins were detected on the membrane with affinity purified TLRR antibody. Immune complexes bound to the membrane were detected with horseradish peroxidase-conjugated donkey secondary antibody (Jackson ImmunoResearch Inc.; West Grove, PA) diluted 1:40,000 in TTBS (100 mM Tris, pH 7.5, 150 mM NaCl, 0.1% Tween20) and developed with enhanced chemiluminescent reagents as described by the manufacturer (Amersham Pharmacia Biotech; Piscataway, NJ).

### Affinity Purification

All use of animals was approved and conducted in accordance with the *Guide for the Care and Use of Agricultural Animals in Agricultural Research and Teaching*. Mouse testis extract was prepared as previously described [13]. Briefly, decapsulated testes from adult mice were homogenized in buffer (10 mM MES, pH 7.65, 1 mM EGTA, 0.5 mM MgCl_2_, 30% glycerol, 0.1% NP40) containing protease inhibitors (10 μM benzamidine, 0.1 mg/ml leupeptin, 0.1 mg/ml aprotinin, 0.1 mg/ml TAME, 3 μM PMSF) and centrifuged two times, first at 100,000 × g and then 130,000 × g, to remove cellular debris generating a high speed supernatant fraction. Protein concentration in tissue lysates was determined by the Coomassie brilliant blue method (Biorad; Hercules, CA).

An affinity purification column was prepared by attaching the KIFC1 targeting peptide identified previously (GKAASGASGASGRAAAIAGRAD; [12]) to a methacrylate resin (UNC Microprotein Sequencing and Peptide Synthesis Facility, Department of Microbiology, University of North Carolina at Chapel Hill) [34]. The 2 ml column was equilibrated in bead binding buffer (25 mM Potassium Phosphate, pH 7.5, 150 mM KCl, 1 mM MgCl_2_) supplemented with protease inhibitors. Approximately 20–50 mg testis lysate in 1.0 ml binding buffer was loaded onto the column and incubated, with gentle mixing, overnight at 4°C. The column was then washed with a total of 10–12 ml buffer and the flow through collected until the OD_280 _had returned to baseline. Bound complexes were eluted from the column in 200 μl fractions first with IgG elution buffer (Pierce; Rockford, IL) followed by IgG elution buffer with 250 mM added NaCl.

Proteins in eluted fraction were precipitated by addition of 1 μl 2% Na Deoxycholate and 5 μl 10% TCA, vortexed for several seconds, and the samples incubated on ice for 30 minutes. Precipitated proteins were pelleted by centrifugation at 20,000 × g for 10 minutes at room temperature and the supernatant removed. Any residual TCA was then removed from the pellet by incubation with 200 μl acetone, followed by incubation at room temperature for about 10 minutes and centrifugation at 20,000 × g, 10 min at room temperature. The supernatant was removed, the acetone wash repeated and the samples air-dried, resuspended in loading buffer and resolved by PAGE. The presence of the TLRR protein in the column fractions was determined by western blot as described above.

### Indirect Immunofluorescence

TLRR was detected in tissue sections essentially as previously described [11]. Testes were obtained from sexually mature mice (29–31 g, CD-1; Charles River, Wilmington, MA) and immersion fixed overnight in 4% paraformaldehyde (PFA) in phosphate buffered saline (PBS, pH 7.4) after piercing of the capsule. The organs were then incubated overnight in 0.5 M sucrose in PBS, placed in cryoprotectant, cut into 10 μm sections, transferred to Vectabond coated slides (Vector Laboratories; Burlingame, CA), quickly dipped in -20°C acetone, and allowed to dry. The sections were washed with PBS and treated with 0.3% Triton X-100 for 15 minutes at room temperature. The tissue was blocked in 2% BSA in TBST (20 mM Tris, pH 7.5, 154 mM NaCl, 2 mM EGTA, 2 mM MgCl_2_, 0.1% Triton X-100) and incubated with TLRR polyclonal antibody diluted 1:50 in TBST (approximately 100 μg/ml).

The TLRR polyclonal antibody was detected with a Texas Red conjugated donkey anti-rabbit IgG secondary antibody (1:100 dilution; Jackson ImmunoResearch Laboratories; West Grove, PA). DNA was stained with DAPI (Invitrogen; Carlsbad, CA) incorporated into Vectashield mounting media (Vector Laboratories; Burlingame, CA). In some experiments, the acrosome was stained with FITC conjugated peanut agglutinin (Sigma-Aldrich; St. Louis, MO) by inclusion in the secondary antibody incubation at a concentration of 20 μg/ml. The intracellular localization of proteins was observed with a Nikon E600 fluorescence microscope fit with appropriate filters and images captured with an Orca II CCD camera (Hamamatsu, Bridgewater, NJ) and analyzed with Metamorph image analysis and acquisition software (Universal Imaging Corporation, Downingtown, PA).

## Authors' contributions

RW conducted the molecular genetic and biochemical characterization of TLRR as well as purification of antibody and immunocytochemistry. AOS conceived, designed and coordinated the study and drafted the manuscript.
